# Evaluation of Aromatic Characteristics and Potential Applications of *Hemerocallis* L. Based on the Analytic Hierarchy Process

**DOI:** 10.3390/molecules29112712

**Published:** 2024-06-06

**Authors:** Yiming Zhou, Wei Yang, Siyi Zhu, Jianan Wei, Xiaoli Zhou, Minglong Wang, Hongxiu Lu

**Affiliations:** 1 School of Perfume and Aroma Technology, Shanghai Institute of Technology, Shanghai 201418, China; zhouymsit@163.com (Y.Z.); zhouxlsit@163.com (X.Z.); 2 Department of Biomedicine and Health Sciences, Shanghai Vocational College of Agriculture and Forestry, Shanghai 201699, China

**Keywords:** *Hemerocallis*, HS-SPME-GC-MS, volatile components, analytical hierarchy process

## Abstract

*Hemerocallis* L. possesses abundant germplasm resources and holds significant value in terms of ornamental, edible, and medicinal aspects. However, the quality characteristics vary significantly depending on different varieties. Selection of a high-quality variety with a characteristic aroma can increase the economic value of *Hemerocallis* flowers. The analytic hierarchy process (AHP) is an effective decision-making method for comparing and evaluating multiple characteristic dimensions. By applying AHP, the aromatic character of 60 varieties of *Hemerocallis* flowers were analyzed and evaluated in the present study. Headspace solid-phase microextraction gas chromatography–mass spectrometry (HS-SPME-GC-MS) was employed to identify volatile components in *Hemerocallis* flowers. Thirteen volatile components were found to contribute to the aroma of *Hemerocallis* flowers, which helps in assessing their potential applications in essential oil, aromatherapy, and medical treatment. These components include 2-phenylethanol, geraniol, linalool, nonanal, decanal, (E)-*β*-ocimene, α-farnesene, indole, nerolidol, 3-furanmethanol, 3-carene, benzaldehyde and benzenemethanol. The varieties with better aromatic potential can be selected from a large amount of data using an AHP model. This study provides a comprehensive understanding of the characteristics of the aroma components in *Hemerocallis* flowers, offers guidance for breeding, and enhances the economic value of *Hemerocallis* flowers.

## 1. Introduction

*Hemerocallis* L. is an important germplasm resource, with more than 83,000 modern cultivars all over the world [1]. Some varieties are not only edible and medicinal [2,3,4], but also consumed as a resource in perfumes, cosmetics, and essential oils. Recently, *Hemerocallis* flowers have been primarily studied for their ornamental characteristics rather than their aroma quality [5]. However, the aromatic volatiles of *Hemerocallis* flowers also enhance the aesthetic value of ornamental plants [6,7,8,9]. The selection of high-quality aromatic plants relies on various indicators. Important indicators for evaluating the plant germplasm resources include the composition of volatile organic compounds, as well as biological properties, economic value, and resistance to biological and abiotic stress [10]. To maximize the industrial value of plants, high-quality varieties with distinctive aromas were selected from a large number of varieties with purpose. *Hemerocallis* flowers have been reported to be rich in nerolidol, linalool, 2-phenylethanol, and 3-furanmethanol [11]. However, the species and content of volatile components vary significantly among different varieties. Additionally, the same volatile components may contribute differently to the aroma characteristics in different varieties [12]. Breeding excellent varieties from *Hemerocallis* flowers is challenging due to the complexity of aroma components. Currently, the research on the classification of hemerocallis flowers based on complex aroma components is limited, and there is a lack of investigation into their aromas in breeding. By constructing a multi-dimensional aroma evaluation index system, the aroma characteristics can be described more comprehensively and accurately, and the reliability and effectiveness of the research can be improved. Therefore, this study employs such a system to classify *Hemerocallis* flowers and select outstanding varieties.

Various analysis models are used for variety breeding under the influence of multiple variables. Among them, the most common one is principal component analysis (PCA), which serves as a multivariate tool to detect correlations between all the breeding lines and VOCs [13]. In addition, grey relational analysis [14] and fuzzy comprehensive evaluation [15] are also utilized to assess the overall quality of varieties based on their traits, aiming to select specific varieties. The analytic hierarchy process (AHP) is one of the most widely used multicriteria decision-making methods [16], as it can determine the most interesting types according to certain criteria. The principle of the AHP is to determine causal factors, arrange and prioritize these factors hierarchically, ensure consistency, and assign priority weights [17]. The AHP model has been applied to make complex multi-objective decisions in ecology and agroforestry, such as identifying potential ecotourism sites, determining community plant types, and designing green land [18,19,20,21]. Similar issues arise in the assessment of aromatic properties of plants. In a typical study, through the analytic hierarchy process (AHP), three major criteria, including adaptability related, ornamental feature-related, and growth habit-related criteria, and eighteen sub-criteria were proposed and constructed. The model was validated on fifteen herbaceous peony cultivars from different latitudes. It could provide a reference for the introduction, breeding, and application of perennials under ever-changing unfavorable climatic conditions [22]. The AHP model screening is more objective and comprehensive compared to screening based solely on volatile components.

At present, few studies have focused on evaluating the aroma of *Hemerocallis* flowers’ germplasm resources. Jiao et al. used HS-SPME-GC-MS to identify the floral fragrance of 46 *Hemerocallis* varieties (7 species, 1 variety, and 38 cultivated varieties) [23]. Zhou et al. used HS-SPME-GC-MS to assess the aroma of *Hemerocallis*, and compared the major aromatic components among 10 different varieties [11]. These studies focused on the analysis of volatile components but did not investigate their role in variety selection. In these studies, the aroma components of selected varieties of *Hemerocallis* flowers were analyzed. However, the selection of the number of *Hemerocallis* flowers was limited, and the aroma evaluation of different varieties of *Hemerocallis* flowers was not combined with the actual application of resources. In addition, traditional breeding goals focus on flower type and color, ignoring flower aroma. The objective of this study was to evaluate 60 scented *Hemerocallis* varieties in accordance with the criteria for detecting functional volatile components. The objective was to use the mathematical analysis method to screen out superior varieties as resources for essential oils, aromatherapy, and ornamentals in healthcare. A group of standards has been constructed to assess the aromas of Hemerocallis flowers in order to guide future germplasm screening, breeding, and the generation of innovative categories. The establishment of this model can target-screen the optimal varieties. By measuring aroma instead of extracting and analyzing the essential oils, this approach can also shorten the duration of the experiment, allowing for quicker and more precise screening using fewer flowers.

## 2. Results

### 2.1. Determination of Volatile Compounds

**Figure 1:**
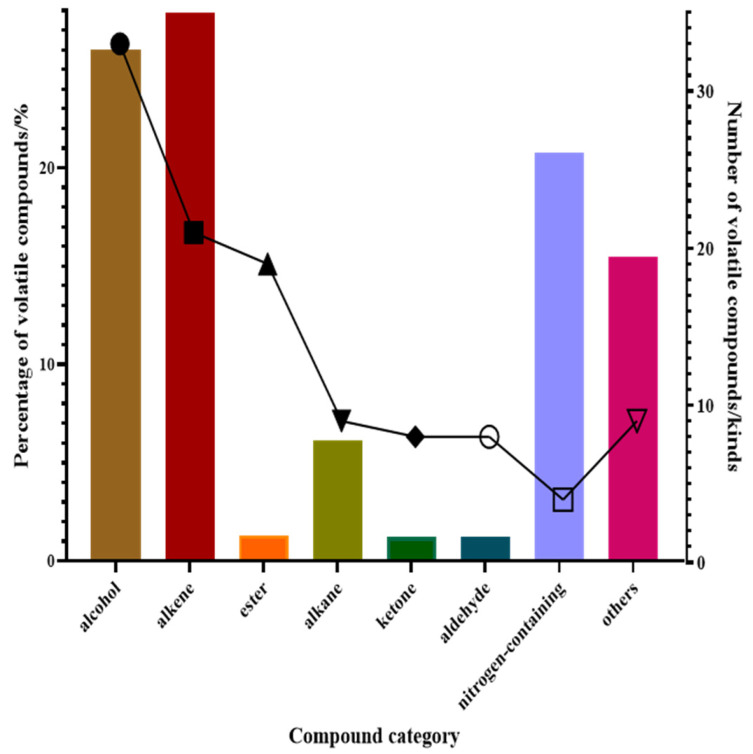
The number and content of compounds in 60 *Hemerocallis* flower varieties.

A total of 114 volatile compounds, mostly alcohols, alkenes, and esters, were identified from flowers of 60 different species of *Hemerocallis* flowers. By categorizing the volatile compounds, an analysis was conducted on the total quantity and content of these compounds, resulting in the determination of their respective proportions in different varieties of *Hemerocallis* flowers. Volatile molecules, including nerolidol, linalool, 2-phenylethanol, nonan-1-ol, and 3-furanmethanol contributed to a large percentage of the alcohol compounds. (E)-*β*-ocimene, α-farnesene, and (+)-α-pinene were the most significant fractions of the alkene compounds. Phenylacetonitrile was the main component in the nitrogen-containing chemicals. These compounds may contribute to the essential components of *Hemerocallis* flowers (Figure 1).

### 2.2. Analysis of Major Floral Aromatic Components

The odor active value (OAV) refers to the ratio of absolute or mass concentration (ρ) of aroma components in an aroma system to its aroma or sensory threshold (T) [24]. By calculating the OAV and referring to the aroma descriptions, 33 important compounds with an OAV greater than 1 were selected out of 114 volatile components. In order to develop an AHP model to evaluate its potential applications in essential oils, aromatherapy, and healthcare, we screened out most of the volatile substances common to 60 species of *Hemerocallis* flowers. To develop an AHP model for evaluating potential applications of essential oils, aromatherapy, and healthcare, most of the volatile compounds common to 60 species of *Hemerocallis* flowers were excluded. 

**Figure 2:**
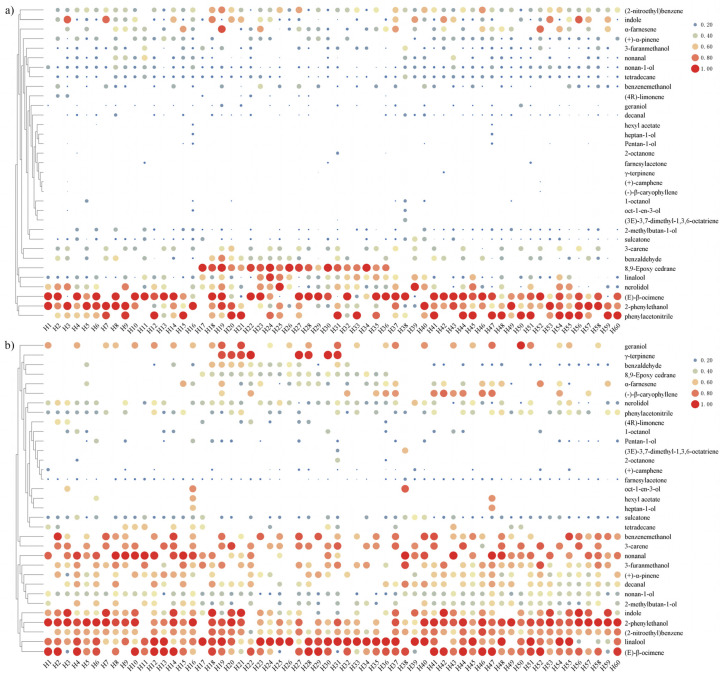
Hierarchical clustering heat maps of 33 main volatile constituents of 60 species of Hemerocallis: (**a**) Concentrations; (**b**) OAVs.

After analyzing the concentration and OAV thermograms of 33 volatile components, it was determined that 13 of these volatile components are present in most varieties. These 13 compounds (OAV) are as follows: 2-phenylethanol (75–2555), geraniol (63.63–1427), linalool (8.33–9191.67), nonanal (45.45–5918.18), indole (5.45–1827.27), 3-furanmethanol (22.22–1031), (E)-*β*-ocimene (1.76–1772.94), benzenemethanol (31.42–1225.45), 3-carene (10.39–789.61), decanal (13.33–376.67), α-farnesene (1.02–167.94), nerolidol (3.28–156.08), and benzaldehyde (1.06–92.50) (Figure 2). These components exhibit s strong aroma, as indicated by their high OAVs. They are the main aromatic substances of most *Hemerocallis* flower varieties. 

### 2.3. Weighted Scores and Resource Evaluation

The weight (*Wi)* values are shown in Table 1 and Table 2; the matrices are satisfactorily consistent. And the *λ*_max_ value implies that the matrices are satisfactorily consistent. 

**Figure 3:**
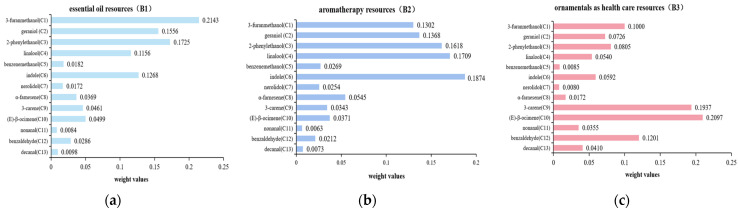
Ranking weights of elements in the scheme hierarchy to decision-making hierarchy: (**a**) essential oil resources (B1); (**b**) aromatherapy resources (B2); (**c**) ornamentals as healthcare resources (B3).

The ranking weight of each element in the scheme hierarchy was calculated as follows: ranking weight of each element in the scheme hierarchy relative to the criteria hierarchy × ranking weight of each element in the corresponding criteria hierarchy relative to the decision-making hierarchy. Combined with the actual score for each element in the scheme hierarchy for each selected variety, the *Wi* value of each element in the scheme hierarchy relative to the decision-making hierarchy was calculated (Figure 3). The best variety was then identified by substituting the volatile components of each *Hemerocallis* flower into the computation.

**Table 1:** Criteria hierarchy–scheme hierarchy judgment matrix for the evaluation of floral fragrance application evaluation.

Floral Fragrance Application Evaluation		C1	C2	C3	C4	C5	C6	C7	C8	C9	C10	C11	C12	C13	*Wi*	
X1	C1	1	2	2	-	-	-	-	-	-	-	-	-	-	0.5000	*λ*_max_: 3 Proportion of consistency: 0
	C2	0.5	1	1	-	-	-	-	-	-	-	-	-	-	0.2500	
	C3	0.5	1	1	-	-	-	-	-	-	-	-	-	-	0.2500	
X2	C2	-	1	0.3333	1	2	0.3333	3	1	-	-	-	-	-	0.1131	*λ*_max_: 7.7206 Proportion of consistency: 0.0883
	C3	-	3	1	0.2	3	0.2	5	3	-	-	-	-	-	0.1526	
	C4	-	1	5	1	5	1	5	5	-	-	-	-	-	0.2698	
	C5	-	0.5	0.3333	0.2	1	0.1667	1	0.3333	-	-	-	-	-	0.0425	
	C6	-	3	5	1	6	1	5	4	-	-	-	-	-	0.2959	
	C7	-	0.3333	0.2	0.2	1	0.2	1	0.3333	-	-	-	-	-	0.0400	
	C8	-	1	0.3333	0.2	3	0.25	3	1	-	-	-	-	-	0.0861	
X3	C9	-	-	-	-	-	-	-	-	1	1	5	3	3	0.3228	*λ*_max_: 5.2837 Proportion of consistency: 0.0633
	C10	-	-	-	-	-	-	-	-	1	1	5	3	5	0.3495	
	C11	-	-	-	-	-	-	-	-	0.2	0.2	1	0.2	1	0.0591	
	C12	-	-	-	-	-	-	-	-	0.3333	0.3333	5	1	5	0.2002	
	C13	-	-	-	-	-	-	-	-	0.3333	0.2	1	0.2	1	0.0684	

**Figure 4:**
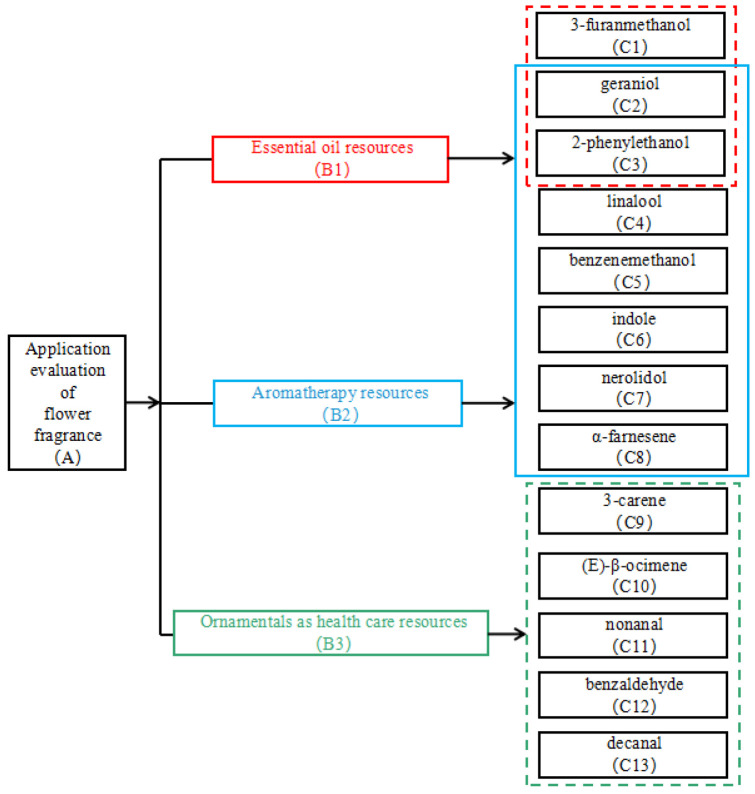
Analytical hierarchy process (AHP) model. The decision, criteria, and scheme hierarchies are presented at the top, middle, and bottom, respectively.

X1: the evaluation of essential oil resources (B1); X2: the evaluation of aromatherapy resources (B2); X3: the evaluation of ornamentals as healthcare resources (B3); C1–C13: the elements in the scheme hierarchy as shown in Figure 4.

**Table 2:** Decision-making hierarchy–criteria hierarchy judgment matrix for the evaluation of floral fragrance application evaluation.

Floral Fragrance Application Evaluation		X1	X2	X3	*Wi*
X1	X1	1	1	3	0.4286
	X2	1	1	3	0.4286
	X3	0.3333	0.3333	1	0.1429
	*λ*_max_: 3; proportion of consistency: 0				
X2	X1	1	0.3333	3	0.2605
	X2	3	1	5	0.6333
	X3	0.3333	0.2	1	0.1062
	*λ*_max_: 3.0387; proportion of consistency: 0.0372				
X3	X1	1	1	0.3333	0.2000
	X2	1	1	0.3333	0.2000
	X3	3	3	1	0.6000
	*λ*_max_: 3; proportion of consistency: 0				

X1: the evaluation of essential oil resources (B1); X2: the evaluation of aromatherapy resources (B2); X3: the evaluation of ornamentals as healthcare resources (B3).

After computing the weighted scores, a comprehensive evaluation was conducted to assess the potential value of 60 *Hemerocallis* flower cultivars as resources for essential oils, aromatherapy, and healthcare.

## 3. Discussion

An AHP model was established based on 13 significant aroma compounds found in the study and these 13 volatile components are present in most varieties. Most alcohols have a sweet aroma. Nerolidol found in high concentrations in “H25” and “H39”, has a sweet fruity flavor with a tinge of woody flavor [25]. 2-phenylethanol has a rose flavor, it was detected in high concentrations in most varieties (“H1”, “H3”, etc.) [26]. The flavor of α-farnesene, which is present in “H19” at high concentration, is fruity and floral [27]. Numerous compounds contribute to the flavor of *Hemerocallis* flowers. The concentrations and thresholds of these compounds vary for the corresponding odor activity values (OAVs). The OAV refers to the ratio of absolute or mass concentration (ρ) of an aroma component in an aroma system to its aroma or sensory threshold (T) [24]. The OAV was applied to properly assess the contribution of each compound to the overall scent of *Hemerocallis* flowers.

Geraniol, benzenemethanol, 3-furanmethanol, 3-carene, nonanal, and decanal despite their lower concentrations, have higher OAVs because of their thresholds. And among most varieties, geraniol has the highest OAV in “H50” and has a fruity fragrance [28]. Benzenemethanol has the highest OAV in “H2”, “H14”, and “H55” and has a rose flavor [29]. 3-furanmethanol also has a sweet aroma and has the highest OAV in “H38” and “H52” [30]. 3-carene has a fruity aroma and has the most significant OAV in “H20” [31]. Nonanal and decanal have fruity and floral flavors that contribute to the aroma of *Hemerocallis* flowers in most varieties [32]; uniquely, there are both high concentrations and a high OAVs of nonanal and decanal in “H38”. The concentration of linalool is highest in “H24”; however, due to its threshold, the OAV is higher in most varieties (“H12”, “H13”, etc.); it has a fruity fragrance [33]. Benzaldehyde contributes a woody flavor to *Hemerocallis* flowers, particularly in “H19” where it is present in high concentrations and high OAVs [34]. (E)-*β*-ocimene has a grassy taste and floral aroma and is commonly found in high concentrations and with a high OAV value in most varieties (“H1”, “H2”, etc.) [35]. The flavor of indole is floral; it is found in high concentrations and high OAVs in “H3”, “H7”, “H18”, “H21”, “H42”, “H53”, and “H56” [36].

The 11 varieties “H2”, “H3”, “H14”, “H19”, “H24”, “H25”, “H38”, “H39”, “H50”, “H52”, and “H55” can be used as the best varieties for aromatherapy as well as essential oil extraction among the 60 varieties. On the other hand, this shows that essential oils are appropriate for aromatherapy. Healthcare resources can use “H13”, “H19”, “H20”, and “H38”.

To better research the perfume of *Hemerocallis*, the flowers of the 13 varieties “H2”, “H3”, “H13”, “H14”, “H19”, “H20”, “H24”, “H25”, “H38”, “H39”, “H50”, “H52”, and “H55” can be selected for further research.

This study focuses on the role of aroma components in breeding *Hemerocallis*. Previous related studies have solely concentrated on the analysis of aromatic components. In Zhou’s study [11], the aroma components of the 10 *Hemerocallis* flower varieties with the largest number of plants and the strongest flavor were analyzed, and the research results were the key odor substances that revealed the aromatic differences of the 10 *Hemerocallis* flower varieties. Jiao and colleagues [23] analyzed the aroma components of 46 kinds of *Hemerocallis* flowers. A total of 37 volatile compounds were identified, including 30 terpenoids, five benzenoids, and two nitrogenous compounds. Terpenoids are the main volatile components that affect the fragrance of *Hemerocallis* flowers. In these studies, the selection of *Hemerocallis* flower varieties was limited, and the aroma evaluation of different varieties of *Hemerocallis* flower was not combined with the practical application of resources. 

In this study, *Hemerocallis* flower varieties with complex fragrance were selected and analyzed. A wide range of *Hemerocallis* flower varieties was chosen and the significant aromas of different *Hemerocallis* flower varieties were evaluated by combining various application resources. The AHP and the multi-index evaluation system were established to quantify the aroma characteristics of these varieties. This evaluation system has potential application value, as it can be used for decision making and improvement in perfume and flower breeding. This will provide a reliable basis for decision making in related fields and promote the development and application of follow-up research.

## 4. Materials and Methods

### 4.1. Plant Materials

**Figure 5:**
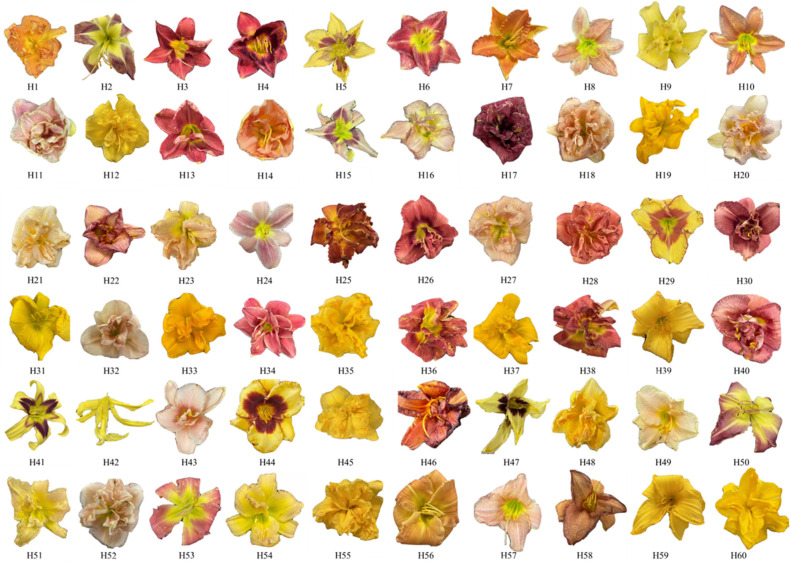
The 60 different varieties of *Hemerocallis.*

In total, 60 species of *Hemerocallis* flowers with strong fragrance were screened from several hundred samples (Figure 5). Sixty distinct varieties of *Hemerocallis* flowers with distinct petals were planted in the experimental garden of the Shanghai Institute of Technology (30°500 N, 121°300 E, 6.67 m above sea level) under identical growing conditions. Three flower samples of each variety were picked.

### 4.2. Detection of Volatile Organic Compounds by HS-SPME-GC-MS

The aroma of different species of the daylilies was analyzed by HS-SPME-GC-MS (Shimadzu, Tokyo, Japan) and semi-quantified by an internal standard method. Fresh *Hemerocallis* flowers (accurately weighed) were placed in headspace containers. After adding 20 μL of 2-octanol (4.47 mg/kg), the lid was covered and the aged SPME (Supelco, Darmstadt, Germany) head was inserted into the collection bottle for adsorption at 60 °C for 90 min [11]. The Shimadzu software (v. 4.45, GCMS Solution, Shimadzu, Tokyo, Japan) was used to process the acquired chromatographic and spectroscopic data. The volatile compounds were identified based on retention times and mass spectra (comparison with the spectra available in the NIST14.L library, similarity greater than 80%) [37]. For the semi-quantitative measurement of compound concentrations, the internal standard method was used. To be more precise, the ratio of the internal standard peak area to the overall peak area was used to calculate the relative concentration of each component.

### 4.3. Evaluation of the Floral Fragrances of 60 Scented Hemerocallis Flower Varieties

Key aroma components were filtered out prior to using AHP to compute the weighted values of 60 *Hemerocallis* flower types. To evaluate each variety, relative objective and unified rating criteria were used. This complete assessment of the tested varieties found those with outstanding comprehensive traits.

#### 4.3.1. Development of the Analytical Hierarchy Process Model

The compositions and concentrations of aromatic components in the 60 types were studied and the data were analyzed by establishing the AHP model (Figure 4). The impacts of different aroma components on emotional stability, slumber, and health-related traits were chosen as the most significant factors in all the types. 

Excellent varieties can be chosen using the AHP methodology. The AHP model used in the current study included three hierarchies. The findings of the assessment of *Hemerocallis* flower varieties in terms of their applications were included in the decision-making hierarchy. The criteria hierarchy included the variables used to assess the viability of each type for use as essential oil resources (B1), aromatherapy resources (B2), and ornamentals as healthcare resources (B3). The scheme hierarchy included statistics for 13 significant aroma compounds (C1–C13). The main volatile substance in the essential oil of daylily is 3-furanmethanol [38]. Geraniol and 2-phenylethanol are important components in many essential oils and have been listed in the international standard for rose oil [39]. Therefore, 3-furanmethanol, geraniol, and 2-phenylethanol were selected as the evaluation factors of essential oil resources (C1–C3).

Aromatherapy is one of the complementary therapies for improving people’s physical and mental health by introducing aromatic substances (also known as essential oils or volatile substances) from plants into the body through fumigation, atomization, massage, sniffing, and other methods. Numerous studies have demonstrated that aromatherapy helps alleviate or heal conditions associated with the neurological system, such as postpartum depression, stress, insomnia, and anxiety [40,41,42]. The composition of Compound Anshen essential oil was analyzed by gas chromatography–mass spectrometry (GC-MS). Among the components, linalool exhibits anti-anxiety and anti-depressant effects [43]. So *Hemerocallis* flowers can be used as an aromatherapy resource.

Linalool has a sedative effect and an inhibitory effect on the central nervous system, while it has good skin absorption properties during massage [44]. Linalool, one of the main components of lavender essential oil, also has an effect on the receptor binding of gamma-aminobutyric acid (GABA). It also increases the expression of dopamine D3 receptor subtypes in the olfactory bulb, increasing the inhibitory intensity of the nervous system, which can have an anti-anxiety effect and is often used as aromatherapy material [45]. Jasmine essential oil is often used in aromatherapy, because it contains linalool, benzenemethanol, 2-phenylethanol, indole, and other aromatic substances, which have physiological activities such as anti-fatigue and anti-oxidation. Several essential oils contain geraniol in high concentration, which can be an anti-depressant [46,47]. Therefore, linalool, benzenemethanol, indole, nerolidol, *α*-farnesene, geraniol, and 2-phenylethanol were selected as the evaluation factors of aromatherapy resources (C2–C8).

Choosing ornamental plants with health benefits refers to choosing cultivars with anti-inflammatory, antibacterial, and antioxidant qualities. In addition to increasing a plant’s resistance to several diseases and herbivorous insects, (E)-*β*-ocimene also inhibits the growth of Escherichia coli and *Staphylococcus aureus*. 3-carene has antibacterial, antioxidant, anticancer, and antiviral properties [48]. Benzaldehyde inhibits Shigella, Salmonella, Escherichia coli, and Staphylococcus aureus to varying degrees [49]. Bacteria and actinomycetes cannot grow when decanal and nonanal are present. Decanal, nonanal, benzaldehyde, 3-carene, and (E)-*β*-ocimene were used as the evaluation criteria for healthcare resource (C9–C13). 

For the following computations, the AHP model was entered into the AHP software (Yaahp Standard Edition, V12.11.8293, Shanxi Yuan Decision Software Technology, Taiyuan, China). Materials with various application values were screened for assessment of different applications by varying the proportional significance of each element in the pairwise comparison in the criteria hierarchy.

#### 4.3.2. Construction of Judgment Matrices 

To make it easier to compare components with varying qualities and to increase accuracy, the factors were compared in pairs using a relative scale. Each factor of each scheme hierarchy beneath a certain criterion level was compared in pairs and ranked according to its significance for that level. In the AHP model, rising levels of importance are shown in numbers 1, 3, 5, 7, and 9. The reciprocal of scales shows that when two elements are compared, the latter is more significant. If required, the median of two neighboring assessments was represented by the numbers 2, 4, 6, and 8. 

These judgment matrices for the resource assessments were created taking into account the significance of scheme hierarchies (factors C1 through C13) in the criteria hierarchy (B1–B3). Similarly, judgment matrices for evaluation of the three resources were developed according to the decisions in the criteria hierarchy (Table 1 and Table 2). The values from the judgment matrices were entered into the AHP program.

#### 4.3.3. Consistency Test and Calculation of Ranking Weights 

After normalization (so that the sum of the elements in the vector is equal to 1), the judgment matrix’s eigenvector, which corresponds to the maximum eigenroot *λ*_max_, is denoted by the letter *Wi*. The ranking weight for the relative importance of a factor at one level to another is called element *Wi*. The term “hierarchical single ranking” refers to this method. For the single hierarchical sort to be confirmed, a consistency test is required. The proportion of consistency (CR) = CI/RI. CI = (*λ*_max_ − n)/(n − 1); CI is the consistency index of the judgment matrix. To measure the size of CI, the random consistency index RI is introduced, RI = (CI1 + CI2 + …+ CIn)/n; RI depends on the order of the judgment matrix. When CR < 0.1, the judgment matrix is considered to have satisfactory consistency. 

To guarantee that each element in the pairwise comparison was consistent, the consistency of the judgment matrices (Table 1) was verified. The judgment matrix was deemed consistent if its highest eigenvalue (max) was equal to or marginally larger than its number of components (n). The evidence was deemed sufficiently reliable. 

Each element’s *Wi* value indicates how significant it is in relation to the element in the level before it. The *Wi* values of the criteria hierarchy and decision-making hierarchy were calculated. The final result of the *Wi* values is related to the total hierarchical sorting. The total hierarchical sorting is used to determine which scheme is best.

The concentration of 13 volatile organic compounds in each variety was used to determine the actual score for each element at the protocol level for each variety. The concentrations of the 13 volatile components in each species were multiplied by their corresponding weight values, then summed. They were then sorted to obtain the optimal varieties. The target materials were evaluated based on the weighted score assigned to each component in the scheme hierarchy. The results list the target materials in order of their scores, which indicates their suitability for each of the three applications. This enables us to find superior types that are appropriate for a range of uses.

## 5. Conclusions

In this study, 114 volatile compounds present in 60 scented *Hemerocallis* varieties were analyzed. Alcohols were detected as the most abundant volatile organic compounds. Nerolidol, linalool, 2-phenylethanol, nonan-1-ol, and 3-furanmethanol were identified as the main volatile organic compounds. The primary aromatic components of *Hemerocallis* flowers were further determined through OAV calculations. 

In conclusion, 2-phenylethanol, geraniol, linalool, nonanal, decanal, (E)-*β*-ocimene, indole, nerolidol, benzaldehyde, 3-furanmethanol, benzenemethanol, 3-carene, and *α*-farnesene contribute to the aroma of *Hemerocallis* flowers. These aromatic chemicals in *Hemerocallis* flowers are the key components in their application in essential oil, aromatherapy, and healthcare.

An AHP model was established based on 13 significant aroma compounds identified in the study, and it was easier to distinguish *Hemerocallis* flowers with complex varieties by this method. This study fully considers the application of aroma in breeding varieties with aromatic properties in complex *Hemerocallis* flowers. The remarkable aroma varieties with aromatherapy and anti-depressant capacities were screened. This work will help improve the chemical understanding of *Hemerocallis* flowers aroma, and promote quality control, breeding, and utilization of *Hemerocallis* flower resources. 

## Data Availability

Data are contained within the article.

## References

[1] Li S., Cui H., Wang J., Hou F., Xing G. (2021). Qualitative and Quantitative Analysis on Flavonoid Distribution in Different Floral Parts of 42 Hemerocallis Accessions. Front. Plant Sci..

[2] Wang D.-Y., Ye Q., Zhang G.-L., Li B.-G. (2003). New anthraquinones from *Gladiolus gandavensis*. J. Asian Nat. Prod. Res..

[3] Mohamed K. (2005). Chemical Constituents of *Gladiolus segetum* Ker-Gawl. Bull. Pharm. Sci. Assiut.

[4] Ngamga D., Awouafack M.D., Tane P., Bezabih M., Abegaz B.M. (2007). Two new anthraquinones from *Gladiolus psittascinus*. Biochem. Syst. Ecol..

[5] Channelière S., Rivière S., Scalliet G., Szecsi J., Jullien F., Dolle C., Vergne P., Dumas C., Bendahmane M., Hugueney P. (2002). Analysis of gene expression in rose petals using expressed sequence tags. FEBS Lett..

[6] Dudareva N., Pichersky E., Gershenzon J. (2004). Biochemistry of plant volatiles. Plant Physiol..

[7] Prasad N.S., Raghavendra R., Lokesh B.R., Naidu K.A. (2004). Spice phenolics inhibit human PMNL 5-lipoxygenase. Prostaglandins Leukot. Essent. Fatty Acids.

[8] Scalliet G., Piola F., Douady C.J., Réty S., Raymond O., Baudino S., Bordji K., Bendahmane M., Dumas C., Cock J.M. (2008). Scent evolution in Chinese roses. Proc. Natl. Acad. Sci. USA.

[9] Atkinson R.G. (2016). Phenylpropenes: Occurrence, Distribution, and Biosynthesis in Fruit. J. Agric. Food Chem..

[10] Gochev V., Wlcek K., Buchbauer G., Stoyanova A., Jirovetz L. (2008). Comparative evaluation of antimicrobial activity and composition of rose oils from various geographic origins, in particular Bulgarian rose oil. Nat. Prod. Commun..

[11] Zhou X., Zhu S., Wei J., Zhou Y. (2023). Volatile metabolomics and chemometric study provide insight into the formation of the characteristic cultivar aroma of Hemerocallis. Food Chem..

[12] Zidi K., Kati D.E., Bachir-bey M., Genva M., Fauconnier M.-L. (2021). Comparative Study of Fig Volatile Compounds Using Headspace Solid-Phase Microextraction-Gas Chromatography/Mass Spectrometry: Effects of Cultivars and Ripening Stages. Front. Plant Sci..

[13] Majithia D., Metrani R., Dhowlaghar N., Crosby K.M., Patil B.S. (2021). Assessment and Classification of Volatile Profiles in Melon Breeding Lines Using Headspace Solid-Phase Microextraction Coupled with Gas Chromatography-Mass Spectrometry. Plants.

[14] Li Y.-Y., Feng Z., Zhao L.-Y., Mo Z.-H., Zhang B. (2007). The Grey Analysis, Kriging and Selection Index of Flower Yield in Rugosa Rose. Agric. Sci. China.

[15] Xiang W., Li K., Dong F., Zhang Y., Zeng Q., Jiang L., Zhang D., Huang Y., Xiao L., Zhang Z. (2023). Development of a multicriteria decision-making model for evaluating hybrid offspring in the sweetpotato (*Ipomoea batatas* L.) breeding process. Breed. Sci..

[16] Wang X., Zhang R., Zhang K., Shao L., Xu T., Shi X., Li D., Zhang J., Xia Y. (2022). Development of a Multi-Criteria Decision-Making Approach for Evaluating the Comprehensive Application of Herbaceous Peony at Low Latitudes. Int. J. Mol. Sci..

[17] Saaty T. (2008). Decision making with the Analytic Hierarchy Process. Int. J. Serv. Sci..

[18] Rahmawaty, Villanueva T., Carandang M. (2011). Participatory Land Use Allocation Case Study in Besitang Watershed, Langkat, North Sumatra, Indonesia.

[19] Rahmawaty, Marpaung R.M.E., Batubara R., Rauf Z. (2022). Analytical hierarchy process (ahp) application in the selection of plant types on the community’s agroforestry land. IOP Conf. Ser. Earth Environ. Sci..

[20] Vaidya O.S., Kumar S. (2006). Analytic hierarchy process: An overview of applications. Eur. J. Oper. Res..

[21] Sewale B., Mammo S. (2022). Analysis of floristic composition and plant community types in Kenech Natural Forest, Kaffa Zone, Ethiopia. Trees For. People.

[22] Feng Y., Cheng X., Lu Y., Wang H., Chen D., Luo C., Liu H., Gao S., Lei T., Huang C. (2022). Gas chromatography-mass spectrometry analysis of floral fragrance-related compounds in scented rose (*Rosa hybrida*) varieties and a subsequent evaluation on the basis of the analytical hierarchy process. Plant Physiol. Biochem..

[23] Jiao F., Liu Q., Sun G.F., Li X.D., Zhang J.Z. (2016). Floral fragrances of *Hemerocallis* L. (daylily) evaluated by headspace solid-phase microextraction with gas chromatography-mass spectrometry. J. Hortic. Sci. Biotechnol..

[24] Zhu Y., Chen J., Chen X., Chen D., Deng S. (2020). Use of relative odor activity value (ROAV) to link aroma profiles to volatile compounds: Application to fresh and dried eel (*Muraenesox cinereus*). Int. J. Food Prop..

[25] Aharoni A., Giri A.P., Verstappen F.W., Bertea C.M., Sevenier R., Sun Z., Jongsma M.A., Schwab W., Bouwmeester H.J. (2004). Gain and loss of fruit flavor compounds produced by wild and cultivated strawberry species. Plant Cell.

[26] Wang Y., Zhang H., Lu X., Zong H., Zhuge B. (2019). Advances in 2-phenylethanol production from engineered microorganisms. Biotechnol. Adv..

[27] Wang X., Cao J., Cheng X., Liu X., Zhu W., Li Y., Wan X., Chen S., Liu L. (2024). UV-B application during the aeration process improves the aroma characteristics of oolong tea. Food Chem..

[28] Li X., Xu Y., Shen S., Yin X., Klee H., Zhang B., Chen K., Hancock R. (2017). Transcription factor CitERF71 activates the terpene synthase gene CitTPS16 involved in the synthesis of E-geraniol in sweet orange fruit. J. Exp. Bot..

[29] Quan W., Jin J., Qian C., Li C., Zhou H. (2023). Characterization of volatiles in flowers from four *Rosa chinensis* cultivars by HS-SPME-GC x GC-QTOFMS. Front. Plant Sci..

[30] Mu L., Tong Q., Liu Y., Meng X., He P., Li G., Ye L. (2023). Application of Gas-Liquid Microextraction (GLME)/GC-MS for Flavour and Fragrance in Ice Cream Detection and Composition Analysis. Molecules.

[31] Sun Q., Wu F., Wu W., Yu W., Zhang G., Huang X., Hao Y., Luo L. (2024). Identification and quality evaluation of Lushan Yunwu tea from different geographical origins based on metabolomics. Food Res. Int..

[32] Wu H., Xu Y., Wang H., Miao Y., Li C., Zhao R., Shi X., Wang B. (2022). Physicochemical Characteristics, Antioxidant Activities, and Aroma Compound Analysis of Seven Peach Cultivars (*Prunus persica* L. Batsch) in Shihezi, Xinjiang. Foods.

[33] Dein M., Munafo J.P. (2022). Characterization of Odorants in White Leaf Mountain Mint, *Pycnanthemum albescens*. J. Agric. Food Chem..

[34] Niu Y., Zhang X., Xiao Z., Song S., Eric K., Jia C., Yu H., Zhu J. (2011). Characterization of odor-active compounds of various cherry wines by gas chromatography-mass spectrometry, gas chromatography-olfactometry and their correlation with sensory attributes. J. Chromatogr. B Analyt Technol. Biomed. Life Sci..

[35] Kim M.K., Lee Y.Y., Lee K.G., Jang H.W. (2019). Instrumental volatile flavor analysis of omija (*Schisandra chinesis* Baillon) using headspace stir-bar sorptive extraction-gas chromatography-mass spectrometry and its relationship to human sensory perceptions. Food Res. Int..

[36] Mindt M., Beyraghdar Kashkooli A., Suarez-Diez M., Ferrer L., Jilg T., Bosch D., Martins Dos Santos V., Wendisch V.F., Cankar K. (2022). Production of indole by *Corynebacterium glutamicum* microbial cell factories for flavor and fragrance applications. Microb. Cell Fact..

[37] Zhou Y., Chen X., Zhu S., Sun M., Zhou X. (2021). Understanding the flavor signature of the rice grown in different regions of China via metabolite profiling. J. Sci. Food Agric..

[38] Lin P., Cai J., Li J., Sang W., Su Q. (2003). Constituents of the essential oil of *Hemerocallis ava* day lily. Flavour. Fragr. J..

[39] Jian H., Feng D., Zhang H., Qiu X., Wang Z., Du W., Xie L., Wang Q., Zhou N., Wang H. (2022). Comparison of Volatile Compounds between Wild and Cultivated Roses. HortScience.

[40] Marchand L. (2014). Integrative and complementary therapies for patients with advanced cancer. Ann. Palliat. Med..

[41] Hwang E., Shin S. (2015). The effects of aromatherapy on sleep improvement: A systematic literature review and meta-analysis. J. Altern. Complement. Med..

[42] Lee S.H., Kim J.Y., Yeo S., Kim S.H., Lim S. (2015). Meta-Analysis of Massage Therapy on Cancer Pain. Integr. Cancer Ther..

[43] Zhong Y., Zheng Q., Hu P., Huang X., Yang M., Ren G., Du Q., Luo J., Zhang K., Li J. (2019). Sedative and hypnotic effects of compound Anshen essential oil inhalation for insomnia. BMC Complement. Altern. Med..

[44] Ali B., Al-Wabel N.A., Shams S., Ahamad A., Khan S.A., Anwar F. (2015). Essential oils used in aromatherapy: A systemic review. Asian Pac. J. Trop. Biomed..

[45] Dobetsberger C., Buchbauer G. (2011). Actions of essential oils on the central nervous system: An updated review. Flavour. Fragr. J..

[46] Mahboubi M. (2016). *Rosa damascena* as holy ancient herb with novel applications. J. Tradit. Complement. Med..

[47] Majdi A., Hosseini S.H., Roozbeh M., Mohammadi A. (2019). Antidepressant and Anxiolytic Effects of Geraniol in Mice: The Possible Role of Oxidative Stress and Apoptosis. Iran. Red Crescent Med. J..

[48] Wang Y., Liu B., Wang X., Fan Y. (2022). Comparison of Constituents and Antioxidant Activity of Above-Ground and Underground Parts of Dryopteris crassirhizoma Nakai Based on HS-SPME-GC-MS and UPLC/Q-TOF-MS. Molecules.

[49] Lee H.H., Ahn J.H., Lee E.S., Kwon A.R., Kwak J.H., Min Y.H. (2014). Chemical Composition and Antimicrobial Activity of the Essential Oil of Apricot Seed. Phytother. Res..

